# Occurrence and Persistence of *Saccharomyces cerevisiae* Population in Spontaneous Fermentation and the Relation with “Winery Effect”

**DOI:** 10.3390/microorganisms12071494

**Published:** 2024-07-21

**Authors:** Alice Agarbati, Francesca Comitini, Maurizio Ciani, Laura Canonico

**Affiliations:** Department of Life and Environmental Sciences, Università Politecnica delle Marche, Via Brecce Bianche, 60131 Ancona, Italy; a.agarbati@univpm.it (A.A.); f.comitini@univpm.it (F.C.); l.canonico@univpm.it (L.C.)

**Keywords:** native *Saccharomyces cerevisiae*, indigenous fermentation, resident yeasts, “winery-effect”, microbial terroir

## Abstract

The yeast *Saccharomyces cerevisiae* ensures successful fermentation in winemaking, although the persistent use of commercial strains lead to the loss of aroma complexity of wines. Hence, the research of indigenous *S. cerevisiae* with proper oenological features and well adapted to specific wine-growing areas become of great interest for winemakers. Here, 206 pure cultures of *S. cerevisiae* were isolated from two wineries during a two-year sampling campaign and bio-typed through interdelta sequences analyses with the aim to evaluate the occurrence and persistence of the *S. cerevisiae* wild population linked to each winery. Both wineries belong to the same Verdicchio DOC wine area (Castelli di Jesi), and never used commercial yeasts during fermentation. Results showed 19 different biotypes with a specific population of *S. cerevisiae* in each winery, without cross-contamination with each other and with commercial starter strains. Moreover, inside each winery a persistence of some dominant biotypes was observed over time (three biotypes in winery 1; 95% of isolates in the two years and one biotype in winery 2; 20% of isolates in the two years), indicating a sort of “winery-effect”. The evaluation of *S. cerevisiae* populations for the oenological characters by microfermentations showed a proper and well distinct aromatic imprinting on the resulted wines supporting the concept of “winery effect”.

## 1. Introduction

*Saccharomyces cerevisiae* represents the yeast mainly responsible for alcoholic fermentation in wine. The use of *S. cerevisiae* commercial active dry wine yeasts drives a vigorous fermentation process linked to a rapid start of fermentation and a complete consumption of fermentable sugars [1], ensuring uniform and constant quality of the wine over the time. On the other hand, pure fermentation could lead the loss of aromatic variability, complexity, and sensorial varietal aroma of wines [2]. Moreover, the widely use of commercial yeasts contrast with the growing interest for organic and natural wines, a new relevant trend in the wine sector. For this reason, a great interest of winemakers is addressed through the use of indigenous *S. cerevisiae* yeasts. They are new yeasts with unknown new genotypes and characterized by a specific secondary metabolite profile. A suitable strategy to detect new *S. cerevisiae* strains is to carry out a sampling campaign of isolation in their natural ecosystem such as vineyard [3] or cellar environments. In the latter case, it is better to choose cellars that never use active dry yeasts to avoid contamination by commercial starters. 

Indigenous yeasts in their natural ecological niches are well adapted to climatic conditions and grape varieties surviving during winemaking practices of a specific wine-growing area [4] with a possible enhancement in the typicity of wines. In this picture, spontaneous wine fermentation conducted by several indigenous *S. cerevisiae* strains, and also indigenous non-*Saccharomyces* yeasts, may be a way to obtain distinctive aromatic profile of wine. It has been widely demonstrated that, although different indigenous *S. cerevisiae* strains are involved in the process, only a few strains represent the dominant population of the fermentation process [5,6,7,8]. This advantage could be related to the highest competitivity of the dominant yeasts to survive under oenological conditions such as increasing ethanol concentrations, low pH values, temperature variations and cell-to-cell interactions with other yeasts [9,10]. Another feature of indigenous yeasts is the so called “winery effect” meaning that some indigenous *S. cerevisiae* strains may persist in the same winery for several consecutive years characterizing the wines of the territory [7,8,11]. This is probably due to the better adaptation of these resident yeasts to a specific winery’s winemaking managements, including climate and agricultural practices. They may play a relevant role in determining organoleptic properties of the wines, which also related to the specific winery [7]. These aspects may reinforce the relationship between the provenience of indigenous *S. cerevisiae* strains and the aroma profile of the resulting wine [12]. On the contrary, Santamaría and co-workers [4] found that there are no representative strains from the winery or the area in the long term (3–4 consecutive years) when investigating the biodiversity of native *S. cerevisiae* in 11 wineries. 

In this work, the occurrence and persistence of *S. cerevisiae* populations of two wineries situated within the Verdicchio DOC winemaking area (distant c.a. 20 Km) was investigated with the aim of increasing knowledge on genetic variability of indigenous *S. cerevisiae* populations in relation to a specific winery, known as the “winery effect”, and their contribution on the typicity of wine. Both wineries never used commercial wine strains, and instead employed spontaneous fermentations of a pre-inoculum (“*pied de cuve*”) that involved collecting grapes a few days before harvest and fermenting must in a reduced volume to induce and control the alcoholic fermentation which is then used to inoculate successive bathes of must. The isolated *S. cerevisiae* strains were bio-typed and oenologically characterized to evaluate the relation among origin, biotype, and oenological characters.

## 2. Materials and Methods

### 2.1. S. cerevisiae Strains Origin and Sampling

**Figure 1:**
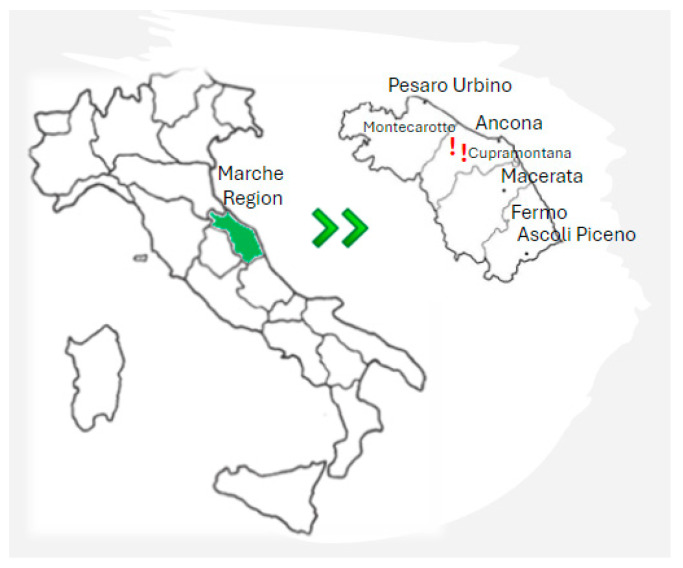
Geolocalization of winery 1 (Cupramontana) and winery 2 (Montecarotto) and both are in Marche Region, Italy.

A total of 206 *S. cerevisiae* isolates, belonging to the microbial collection of the Dipartimento di Scienze della Vita e dell’Ambiente (DiSVA), Università Politecnica delle Marche (Italy), were previously isolated from two different wineries, located 20 km away from each other. The first cellar (winery 1) was in the town of Cupramontana (coordinates: 43.45939, 13.12515), the second cellar (winery 2) was in Montecarotto (coordinates: 43.532023, 13.075781) (Figure 1), and both in the winemaking area of Verdicchio DOC Castelli di Jesi Marche region, Italy. Ninety-nine *S. cerevisiae* strains came from winery 1, while 107 strains came from winery 2. These strains were collected during a two-year sampling campaign.

In winery 1, 43 isolates were collected in the year 2016 and 56 isolates in the year 2019. The yeasts were isolated from grapes, after 7/15 days of auto-enrichment, during “*pie de cuve*”, tank fermentation, winery equipment and winery environment. Regarding winery 2, 80 isolates were collected in the year 2021 and 27 isolates in the year 2022. The isolates came from the winery work equipment and environment, fermentation tanks and during “*pie de cuve*”.

The sampling campaign was carried out by collecting about 1 Kg of undamaged ripe grape bunches in sterile plastic bags, and 50 mL of fermenting must in sterile tubes from “*pie de cuve*” must fermentation samples. Winery equipment and environment samples were performed using sterile swabs streaked on random surfaces (10 cm^2^). All the samples were stored on ice during sampling and transported to the laboratory as soon as possible for their processing and *S. cerevisiae* isolation and identification [8].

The wineries did not use commercial starter strains.

### 2.2. Genotyping Characterization of S. cerevisiae Strains

All *S. cerevisiae* strains were subjected to genotyping through interdelta analyses, using primer pairs delta12 (5′-TCAACAATGGAATCCCAAC-3′)/delta21 (5′-CATCTTAACACCGTATATGA-3′) to analyze the interdelta sequence, as described by Legras and Karst [13]. The genomic DNA of yeasts was extracted at 95 °C for 10 min using a Biorad Thermal Cycler [14] and used to carried out the amplification as follows: 3 min at 95 °C, then 25 s at 94 °C, 30 s at 45 °C, and 90 s at 72 °C for 9 cycles and 25 s at 94 °C, 30 s at 50 °C, and 90 s at 72 °C for 21 cycles. A final extension was performed at 72 °C for 10 min. Amplicons obtained were separated by electrophoresis on 1.5% (*w*/*v*) of agarose gel submitted to 66 V for 1.5 h in 0.5× TBE buffer, to compare the electrophoretic profile of yeasts between them. Different profiles obtained using the interdelta analyses defined the different biotypes. Four commercial starter strains, commonly used in Verdicchio wine production, were used to exclude any commercial contamination: three strains of *S. cerevisiae*, Lalvin EC1118, Lalvin ICV OKAY^®^ (Lallemand Inc., Toulouse, France) and VIN13 (Anchor Wine Yeast, Cape Town, South Africa), and a strain of *Saccharomyces bayanus* N96 (Anchor Wine Yeast, Cape Town, South Africa).

### 2.3. Analyses of Biotypes for H_2_S Production and Killer Activity

Biotypes were tested for their ability to produce H_2_S (hydrogen sulphide), an undesirable compound in wine (confers rotten egg aromatic note), following the procedure reported by Agarbati et al. [15]: pure culture of each biotype was spread on a BiGGY agar medium (Oxoid Ltd., Cheshire, UK) and incubated at 25 °C for 48 h. In this medium, the colonies H_2_S-negative appear white, while those H_2_S-positive appear brown/black. For each biotype, a scale from zero (white colony) to 5 (brown/black colony) was used.

Biotypes were also tested for their killer activity, as described by Comitini et al. [16]: a sensitive *S. cerevisiae* strain, DiSVA 42, was seeded into a Malt agar medium (Biolife, Monza, Italia) buffered to pH 4.2 with 0.1 M citric acid/dibasic sodium phosphate, at a final concentration of 10^6^ CFU/mL. Pure cultures of biotypes were spotted on the Malt agar surface and after 48 h of incubation at 25 °C, the strains were designated as killer when there was a clear zone of growth inhibition of the sensitive yeast around the spot.

### 2.4. Fermentation Trials

Biotypes of both wineries were oenologically characterized carrying out microfermentations using Verdicchio grape juice. Freshly harvested grapes were treated following the standard winemaking procedure: soft pneumatic pressing, and cold clarification at 10 °C for 48 h without SO_2_ addition. The analytical characters of the grape juice were as follows: initial sugar content 236 g/L, pH 3.22, total acidity 4.42 tartaric acid g/L, malic acid 2.70 g/L, total SO_2_ 35 mg/L, and yeast assimilable nitrogen (YAN) content 89 mgN/L. The YAN was adjusted to 250 mgN/L by the addition of diammonium phosphate and yeast derivative (Genesis Lift^®^ Oenofrance, Bordeaux, France). Then, the grape juice was treated overnight at 4 °C with 0.2 mL/L of dimethyl dicarbonate to suppress the wild yeasts. This was confirmed through viable cell counts using WL nutrient agar (Wallerstain Laboratories, Oxoid, Hampshire, UK). Biotypes were precultured in modified YPD (0.5% *w*/*v* yeast extract, 2% *w*/*v* glucose, and 0.1% *w*/*v* peptone) for 24 h at 25 °C, washed twice with sterile water and used to inoculate (c.a. 1 × 10^6^ cells/mL) flasks containing 70 mL of Verdicchio grape juice. The flasks were locked with a Pasteur hydraulic valve, incubated at 22 °C under static conditions and monitored daily by weight loss, due to the CO_2_ evolution, until the end of the fermentation (constant weight for almost two consecutive days). The trials were carried out in duplicate and *S. cerevisiae* Lalvin ICV OKAY^®^ and *S. bayanus* N96 were used as controls. 

### 2.5. Main Analytical Characters and Volatile Compounds of Microfermentation Trials

The fermentation rate was calculated as g CO_2_/day over the 3rd day of fermentation. Volatile acidity, total acidity, ethanol content, pH and Sulfur dioxide were determined following the methods reported by the International Organization of Vine and Wine [17,18,19,20,21]. Enzymatic kits (Megazyme International Ireland) were utilized to determine glucose and fructose (K-FRUGL) and malic acid (K-DMAL) following the manufacturer procedures. The ammonium content was determined using a specific enzymatic kit (kit no. 112732; Roche Diagnostics, Germany) while the free α-amino acids were evaluated following the Dukes and Butzke protocol [22]. Acetaldehyde, ethyl acetate, n-propanol, isobutanol, amyl- and isoamyl alcohols were determined by direct injection of the sample in a gas chromatograph (GC-2014, Shimadzu, Kyoto, Japan) following the procedure of Canonico et al. [23]. Other volatile compounds such as hexanol, β-phenyl ethanol, isoamyl acetate, phenyl ethyl acetate, ethyl hexanoate, ethyl butyrate, ethyl octanoate, diethyl succinate, ethyl acetate, nerol, geraniol, linalol and β-damascenone were determined using the solid phase microextraction (HS-SPME) approach: 5 mL of the sample was placed into a vial with 1 g of NaCl and 1.6 mg/L of 3-octanol as the internal standard. The sample was stirred for 10 min at 25 °C and placed at 40 °C for 30 min, inserting the fiber Divinylbenzene/Carboxen/Polydimethylsiloxane (DVB/CAR/PDMS) into the vial headspace. Then, the fiber was inserted into a Shimadzu gas chromatograph GC injector, in split–splitless modes, using a glass capillary column of 0.25 µm Supelcowax 10, length 60 m, and internal diameter 0.32 mm, to allow the desorption of the compounds [24].

### 2.6. Data Analyses

The experimental data regarding the fermentation rate, volatile acidity and ethanol were subjected to analysis of variance (ANOVA) through Duncan’s tests, considering an associated *p*-value < 0.05 (software STATISTICA 7, Statsoft inc., Tulsa, OK, USA). The mean values of by-products of fermentation and volatile compounds were used to carry out Principal Component Analysis (PCA) using JMP 11^®^ statistical software (Statistical discovery from SAS, New York, NY, USA).

## 3. Results

### 3.1. Genotyping, Occurrence, H_2_S Production and Killer Activity of S. cerevisiae Strains

**Figure 2:**
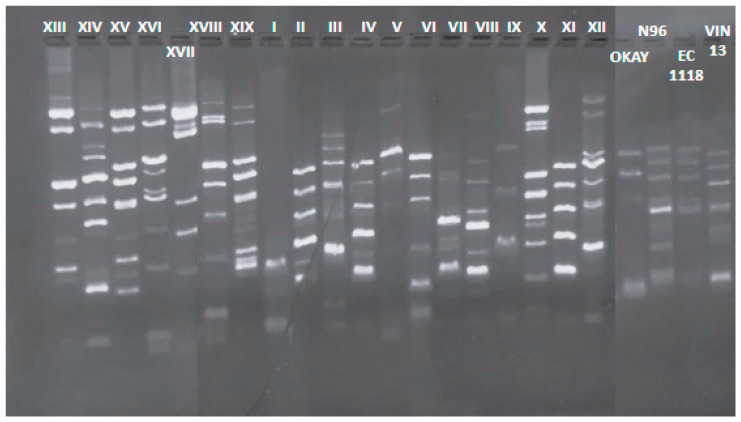
Different interdelta profiles of biotypes of both wineries. Each biotype is represented as a Roman numeral, from XIII to XIX for winery 1 and from I to XII for winery 2. All biotype’s profiles were compared with those of commercial strains OKAY^®^, N96, EC1118 and VIN13.

Interdelta sequence analyses of the 206 *S. cerevisiae* isolates (99 winery 1 and 107 winery 2) revealed a total of 19 different biotypes, as showed in Figure 2. Seven different biotypes had been found in winery 1, and 12 different biotypes in winery 2, highlighting more biodiversity in the lastly winery. Biotypes from the two wineries were different and both biotype groups were different from the more diffused commercial strains utilized in the Verdicchio wine area and used as reference.

Regarding the occurrence of each biotype within the winery, in both there are two biotypes that represent the dominant population, biotype XIII and XIV in winery 1 and biotype I and IV in winery 2 (Table 1). In winery 1, the two biotypes represented more than 90% of the entire population of *S. cerevisiae* strains while the occurrence of each of the other biotypes was ≤2%. Moreover, both dominant biotypes and the biotype XVI had been found in both two-year sampling campaigns, as “resident yeasts” (95%). Indeed, persistent biotypes XIII and XIV represented 68% and 19%, respectively, of the *S. cerevisiae* population detected in 2016 while the same biotypes represented 42% and 43%, respectively, of the *S. cerevisiae* population detected in 2019.

In winery 2, a wide biotype’s variability was observed. The dominant biotypes represented c.a. 40% of the *S. cerevisiae* population, followed by the biotype III (c.a. 17%) and biotype II (15%). The remaining 28% of the population was grouped in the other biotypes. Similarly to winery 1, in winery 2 a resident biotype (biotype IV) was also found that represented 18% and 6% of the *S. cerevisiae* population detected in 2021 and 2022, respectively.

The results (Table 1) show a low or absent production of H_2_S (rotten egg aroma) for all biotypes of both wineries. All of them showed H_2_S production with a score ≤ 2.0. The evaluation of the presence of a killer phenomenon showed that there was a wide diffusion among the biotypes. Indeed, all biotypes of both wineries showed killer activity against the sensitive *S. cerevisiae* yeast DiSVA 42, with the only exception of the biotype II belonging to winery 2 (Table 1).

### 3.2. Oenologically Characterization

#### 3.2.1. Fermentation Rate, Volatile Acidity and Ethanol Production of Biotypes

Results of the main oenological characters of the biotypes of both wineries are reported in Table 2 (more details in Table S1 of Supplementary Materials). No significant differences were detected regarding fermentation rate, volatile acidity and ethanol production comparing the wineries between them and with the commercial starters. Nonetheless, greater variability in the fermentation rate was observed, ranging from 0.9 (Okay ^®^) to 1.4 (winery 1) gCO_2_/day.

#### 3.2.2. Main By-Products of Fermentation

Main by-products of fermentation, responsible for the aromatic profiles of the wines, are reported in Table 3. Overall, a variable production of these compounds is observed among the samples. Nevertheless, *S. cerevisiae* biotypes of winery 1 and winery 2 differ from both commercial strains for the higher production of β-phenyl ethanol and phenyl ethyl acetate that are able to confer a rose aroma, and ethyl hexanoate responsible for a refreshing fruity aroma. On the contrary, they showed lower production of amyl alcohol, isoamyl alcohol (solvent aroma) and diethyl succinate (raspberry and apple aroma) than commercial strains. Main by-products of fermentation produced by each biotype of each winery are reported in Table S2 of Supplementary Materials.

**Figure 3:**
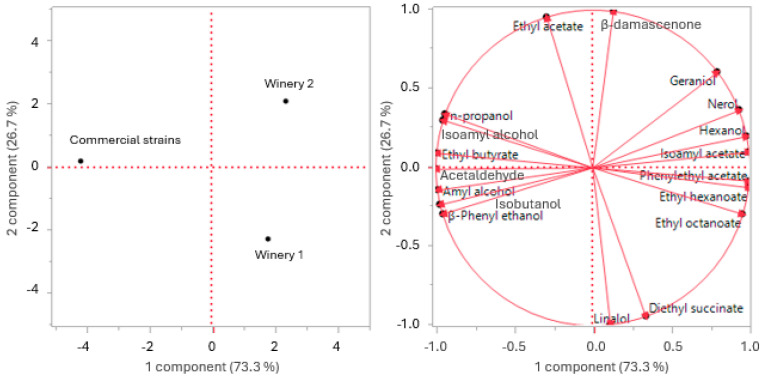
Principal component analysis (PCA) based on the data for the main by-products of fermentation of wines obtained by all biotypes belonging to the two wineries, compared with commercial starter strains.

Results of the main by-products of fermentation were also elaborated through principal component analysis (PCA) to evaluate the clear distinction between wineries and commercial starters (Figure 3) on aroma compounds production. The elaboration explained a total variability of 100% (PCA 1 73.3%; PCA2 26.7%). On the basis of the compounds evaluated, the wineries had well separated each other and from the commercial strains. Winery 1 was distributed in the lower right quadrant and mainly characterized by ethyl octanoate and diethyl succinate production. Winery 2 was in the upper right quadrant and mainly characterized by terpens and isoamyl acetate while commercial strains were placed in the upper left quadrant.

## 4. Discussion

The concept of “terroir”, that refers to wines with distinctive features linked to the characteristics of the geographical area of production, is defined by several characteristics such as the cultivar of the fermented grapes, geographical factors (climate, soil geology and pedology) and the agronomic approach used. This concept was also extended to the biotic factors, such as grape’s microbial communities and the native yeasts involved in the fermentation process, including indigenous *S. cerevisiae* population naturally colonizing cellars, enhancing the aroma qualities of the wine-production area [25]. However, the occurrence and effects of regional/winery specific microbiota in defining wine characteristics are more controversial issues [26,27].

Several studies using high throughput sequencing technologies described microbial communities of grapes and related wines and winery, revealing a link between grape must and soil microbial communities, as well as the geography of the territory [28,29,30]. Other studies showed that there are no specific strains representative of a winery or a winemaking area [4,31,32]. In addition, two vineyards can share a very similar yeast consortium at the genus or species level but may be very different at the strain level since the strong strain’s differences may produce very different wines [33,34,35]. Based on these observations, the concept of “microbial terroir”, although its meaning seems more complex, involves many factors, related to both the plant and the physical environment. Based on this, terroir must be considered by a pluri-disciplinary approach.

Another aspect regarding the concept of “winery effect” are the overall selective factors as winery environment, fermented grapes, and winemaking methodologies. Rosini [36] showed that in a new winery after the initial addition of selected *S. cerevisiae,* it is able to impose itself on the spontaneous fermentation carried out the next year. In another work [37], the selectivity of the winery environment (“winery effect”) was detected between the *S. cerevisiae* population isolated from a modern, working winery, and an abandoned one (since 1914). The *S. cerevisiae* yeasts population from a modern winery showed higher values for characters typically subjected to selective pressure (fermentation power, fermentation rate, SO_2_ resistance). Today, there still remains a lacking concern in the origin and colonization of *S. cerevisiae.* It is very rare on grape and vineyard but was found on damaged grapes [38]. It drives spontaneous fermentation carried out in sterilized vessels [39] and it is also widely found colonizing winery surface and winery equipment (also in a newly built winery) for several years [40,41]. 

In the present work, the *S. cerevisiae* populations of two wineries located in the same winemaking area (Verdicchio DOC Castelli di Jesi) were evaluated with the same methods of conducting fermentation (never used commercial strains, use of spontaneous fermentations with pre-inoculum). A distinct and stable *S. cerevisiae* yeast population was found in both wineries. Some specific genotypes were dominant in each winery in two years (95% and 20% of isolates in winery 1 and winery 2, respectively) confirming the results reported by other authors [7,11,42]. These results may be an additional support for the so called “winery effect” or a microbial terroir at a smaller scale as suggested by other works [7,42,43,44] where some predominant *S. cerevisiae* strains persisted in different fermentations in the same winery from one year to another and they seemed to be representative of a single winery rather than of an oenological area. Moreover, in agreement with Stefanini et al. [45], the results of the PCA analysis of oenological characters of the *S. cerevisiae* population showed distinctive aromatic profiles in each winery population suggesting the possible existence of a winery-specific “microbial-terroir” that may contribute to the final product. However, the variability due to the harvest (“year effect”) can also play an important role in the presence and variability of the biotypes and ultimately on their contribution to the final product. Seasonality and therefore the variability of the characteristics of the grapes and different winery managements may determine the composition of the *Saccharomyces* population.

On the other hand, to have a more comprehensive picture of the influence of specific yeast populations at the winery or at a regional level, further investigations on the microbiota carried out at the strain level and in more different years will be necessary. In this regard, the oenological involvement of non-*Saccharomyces* could also be useful to clarify the picture of specific biotic components on the specific winery and ultimately on “terroir”. 

Non-*Saccharomyces* starters are increasingly used in combination with conventional starters. This consideration leads to a growing interest in the social life of wine yeasts, to better understand the different species of wine yeasts and their ecological context [46,47]. In the short term, the development of multispecies starter cultures and the study of interactions between wine microorganisms are also likely to become key drivers of wine innovation.

## 5. Conclusions

The role of *S. cerevisiae* in winemaking is necessary for a successful wine quality, avoiding stuck fermentations and consequently unpleasant aromatic notes. The exploitation of the indigenous *S. cerevisiae* population linked to a specific winery seems to have become a suitable strategy for winemakers to obtain new *S. cerevisiae* strains as starters of fermentation that are able to confer a diversification of wines in relation to the wine-producing area. Moreover, this study reinforces the concept of the winery-effect in the *S. cerevisiae* population selection and colonization, as resident yeasts. Further investigations over time will be necessary to determine if these yeasts are long-term or short-term resident yeasts.

## Figures and Tables

**Table 1:** Biotypes occurring in wineries, their frequency and characterization in the H_2_S production and killer activity. H_2_S production was expressed as a score, using a hedonic scale from zero (no H_2_S production) to 5 (maximum level of H_2_S production). The presence of killer activity was expressed as “+”, the absence with “−”, and “±” indicates faint killer activity.

Winery	Biotype	Frequency (%)	H_2_S Production	Killer Activity
1	XIII *	49.5	2.0	±
	XIV *	43.5	0.0	+
	XV	1.0	1.5	+
	XVI *	2.0	0.0	+
	XVII	1.0	0.5	+
	XVIII	1.0	0.5	±
	XIX	2.0	0.8	+
2	I	23.4	0.5	+
	II	15.0	0.0	−
	III	16.8	0.0	+
	IV *	19.6	1.0	+
	V	2.8	0.5	±
	VI	7.5	0.5	+
	VII	1.9	1.5	±
	VIII	1.9	0.8	±
	IX	3.7	0.0	±
	X	0.9	1.0	+
	XI	5.6	1.0	+
	XII	0.9	1.0	±

* Biotypes found in both sampling years within the same winery.

**Table 2:** Fermentation rate of biotypes and volatile acidity and ethanol content of resulted wines. Biotypes of the same winery were analyzed as a unique population reported as winery 1 and winery 2 and compared with both commercial starter strains, OKAY^®^ and N96. Data are reported as mean value ± standard deviation and the Duncan’s test (*p*-value < 0.05) reported as different superscript letters (^a,b,c^) within each column represent significant differences.

Strains Population	Fermentation Rate (gCO_2_/day) *	Volatile Acidity (Acetic Acid g/L)	Ethanol (% *v/v*)
Winery 1	1.4 ± 0.2 ^a^	0.4 ± 0.1 ^a^	12.8 ± 0.3 ^a^
Winery 2	1.2 ± 0.1 ^ab^	0.5 ± 0.1 ^a^	12.4 ± 0.3 ^a^
OKAY^®^	0.9 ± 0.0 ^c^	0.4 ± 0.0 ^a^	12.4 ± 0.0 ^a^
N96	1.1 ± 0.0 ^bc^	0.5 ± 0.0 ^a^	12.4 ± 0.1 ^a^

* Fermentation rate calculated at the 3rd day of fermentation.

**Table 3:** Main by-products of fermentation of wines obtained by biotype’s populations of winery 1 and winery 2 compared with those obtained by commercial starter strains, OKAY^®^ and N96. Data are reported as mean value ± standard deviation and Duncan’s test (*p*-value < 0.05) reported as different superscript letters (^a,b,c^) within each row represent significant differences.

Fermentation By-Products	Winery 1	Winery 2	OKAY^®^	N96
Alcohols				
Hexanol (mg/L)	11.84 ± 0.99 ^a^	12.66 ± 1.77 ^a^	10.38 ± 0.61 ^ab^	9.13 ± 0.74 ^b^
β-Phenyl ethanol (mg/L)	7.48 ± 1.12 ^a^	7.39 ± 1.11 ^a^	7.96 ± 0.03 ^b^	7.32 ± 0.00 ^b^
n-propanol (mg/L)	12.60 ± 8.26 ^b^	16.88 ± 2.96 ^b^	42.70 ± 0.09 ^a^	13.51 ± 0.42 ^b^
Amyl alcohol (mg/L)	7.28 ± 4.44 ^b^	4.85 ± 2.39 ^b^	15.42 ± 0.39 ^a^	15.55 ± 0.38 ^a^
Isoamyl alcohol (mg/L)	36.96 ± 10.7 ^b^	41.60 ± 12.07 ^b^	52.01 ± 0.17 ^ab^	60.78 ± 0.37 ^a^
Isobutanol (mg/L)	5.22 ± 3.94 ^a^	8.24 ± 1.78 ^a^	5.29 ± 0.03 ^a^	7.89 ± 0.04 ^a^
Carbonyl compounds				
Acetaldehyde (mg/L)	15.59 ± 9.85 ^b^	12.21 ± 2.06 ^b^	15.05 ± 0.47 ^b^	80.97 ± 0.23 ^a^
Esters				
Isoamyl acetate (mg/L)	0.80 ± 0.39 ^ab^	0.90 ± 0.20 ^a^	0.39 ± 0.00 ^b^	0.35 ± 0.01 ^b^
Phenyl ethyl acetate (mg/L)	0.47 ± 0.16 ^a^	0.47 ± 0.09 ^a^	0.21 ± 0.01 ^b^	0.38 ± 0.02 ^ab^
Ethyl hexanoate (mg/L)	0.65 ± 0.19 ^a^	0.63 ± 0.22 ^a^	0.39 ± 0.00 ^ab^	0.16 ± 0.01 ^b^
Ethyl butyrate (mg/L)	0.03 ± 0.03 ^a^	0.03 ± 0.05 ^a^	0.06 ± 0.00 ^a^	0.05 ± 0.01 ^a^
Ethyl octanoate (µg/L)	3.75 ± 1.04 ^a^	3.60 ± 1.13 ^a^	3.45 ± 0.34 ^a^	2.78 ± 0.05 ^a^
Diethyl succinate (mg/L)	0.04 ± 0.01 ^b^	0.03 ± 0.01 ^b^	0.01 ± 0.00 ^c^	0.06 ± 0.00 ^a^
Ethyl acetate (mg/L)	10.16 ± 3.34 ^b^	11.61 ± 4.61 ^ab^	17.62 ± 0.20 ^a^	5.13 ± 0.03 ^b^
Terpenes				
Linalol (µg/L)	17.85 ± 8.96 ^a^	7.12 ± 5.04 ^a^	8.89 ± 0.53 ^a^	12.51 ± 1.07 ^a^
Nerol (µg/L)	3.41 ± 1.48 ^ab^	4.56 ± 1.50 ^a^	1.95 ± 0.50 ^b^	1.88 ± 0.39 ^b^
Geraniol (µg/L)	4.71 ± 1.58 ^a^	5.69 ± 1.53 ^a^	4.02 ± 0.57 ^a^	4.49 ± 0.47 ^a^
Enones				
β-Damascenone (µg/L)	4.56 ± 2.05 ^a^	4.15 ± 1.41 ^a^	5.89 ± 0.34 ^a^	4.98 ± 0.28 ^a^

## Data Availability

The original contributions presented in the study are included in the article/Supplementary Materials, further inquiries can be directed to the corresponding author.

## Supplementary Materials

The following supporting information can be downloaded at: https://www.mdpi.com/article/10.3390/microorganisms12071494/s1, Table S1: Fermentation rate, volatile acidity and ethanol production of biotypes of winery 1 and winery 2 compared with the data of commercial starter strains OKAY^®^ and N96. Table S2: Main by-products of fermentation of wines obtained by all biotypes of winery 1 and winery 2 compared with those obtained by commercial starter strains OKAY^®^ and N96.
