# Supplementary material for: Occurrence and Persistence of Saccharomyces cerevisiae Population in Spontaneous Fermentation and the Relation with “Winery Effect”
Source: Microorganisms. 2024 Jul 21;12(7):1494. doi: 10.3390/microorganisms12071494 (PMC11278986; doi:10.3390/microorganisms12071494)
Supplement: Supplementary file 1 [file microorganisms-12-01494-s001.zip › microorganisms-3052353-supplementary.pdf]

Table S1. Fermentation rate, volatile acidity and ethanol production of biotypes of winery 1 and winery 2 compared.

Data are reported as mean value ± standard deviation**.

	Biotypes	Fermentation rate (gCO_2/Day)*	Volatile acidity (acetic acid g/L)	Ethanol (% *v*/*v*)
Winery 1	XIII	1.5±0.1	0.6±0.1	12.3±0.1
	XIV	1.4±0.0	0.6±0.0	12.5±0.2
	XV	1.4±0.0	0.4±0.0	12.6±0.2
	XVI	1.5±0.4	0.3±0.1	12.9±0.3
	XVII	1.6±0.1	0.4±0.0	12.9±0.2
	XVIII	1.3±0.0	0.4±0.0	13.1±0.3
	XIX	1.2±0.2	0.6±0.0	12.9±0.0
Winery 2	I	1.2±0.1	0.6±0.0	12.6±0.0
	II	1.0±0.1	0.5±0.1	12.5±0.1
	III	1.3±0.0	0.5±0.1	12.4±0.0
	IV	1.3±0.0	0.6±0.0	12.3±0.0
	V	1.3±0.0	0.4±0.0	12.5±0.1
	VI	1.3±0.0	0.5±0.0	12.4±0.1
	VII	1.3±0.0	0.4±0.1	12.4±0.0
	VIII	1.4±0.1	0.5±0.0	12.6±0.0
	IX	1.1±0.2	0.5±0.0	11.8±0.1
Commercial strains	OKAY®	1.0±0.0	0.4±0.0	12.3±0.0
	N96	1.1±0.0	0.5±0.0	12.4±0.1

*Fermentation rate calculated at the 3rd day of fermentation.

** Biotypes X XI and XII were not evaluated due to the lost during the recovery after conservation.

Table S2. Main by-products of fermentation of wines obtained by all biotypes of winery 1 and winery 2 compared with those obtained by commercial starter strains OKAY® and N96**. Data are reported as mean value ± standard deviation*.

Fermentation by-products	Winery 1 biotypes							Winery 2 biotypes								
**Alcohols**	XIII	XIV	XV	XVI	XVII	XVIII	XIX	I	II	III	IV	V	VI	VII	VIII	IX
Hexanol (mg/mL)	10.43±0.00	12.99±0.68	12.50±0.80	12.43±2.13	11.36±0.71	10.89±0.08	11.17±0.74	10.57±1.32	14.11±1.00	14.07±1.13	16.05±0.72	11.59±1.84	12.03±0.97	12.14±0.01	12.51±0.51	10.82±0.40
β-Phenyl ethanol (mg/mL)	6.38±0.00	8.81±1.10	9.28±1.84	7.27±0.53	8.03±0.03	6.87±0.45	6.21±0.12	6.95±0.18	5.53±0.81	8.14±1.24	8.82±0.59	6.24±0.45	6.54±0.32	8.01±0.55	8.10±0.31	8.20±0.08
n-propanol (mg/mL)	18.32±0.17	13.69±0.72	15.26±0.40	13.29±0.78	21.69±0.35	ND**	ND	16.47±0.32	17.13±0.14	11.62±0.63	14.85±0.18	22.42±0.39	17.71±0.34	16.91±0.30	19.17±0.07	15.59±0.08
Amyl alcohol (mg/mL)	5.20±0.37	0.44±0.63	2.94±0.06	10.96±0.67	6.61±0.37	12.62±0.27	12.32±0.06	9.64±0.94	3.56±0.03	1.53±0.34	4.82±0.09	6.32±0.46	4.14±0.31	4.79±0.09	6.29±0.02	2.53±0.06
Isoamyl alcohol (mg/mL)	40.05±1.46	19.28±0.16	32.96±0.64	26.47±0.31	49.76±0.74	47.33±0.59	45.60±0.67	43.03±0.42	38.15±0.01	19.66±0.56	55.58±0.53	49.26±0.33	50.24±0.05	39.85±0.28	52.08±0.28	26.48±0.11
Isobutanol (mg/mL)	7.67±0.49	5.60±0.20	4.12±0.17	4.52±0.39	10.90±0.50	ND	ND	7.81±0.29	5.42±0.31	8.45±0.40	10.67±0.22	10.49±0.30	6.96±0.16	8.35±0.23	9.49±0.43	6.51±0.22
**Carbonyl compounds**																
Acetaldehyde (mg/mL)	10.09±0.97	4.32±0.27	18.73±1.26	9.27±0.19	14.17±0.53	9.58±0.12	34.70±0.51	14.17±0.49	14.96±0.23	12.68±0.02	11.25±0.17	11.45±0.42	8.09±0.11	11.74±0.41	13.95±0.22	11.55±0.35
**Esters**																
Isoamyl acetate (mg/mL)	1.06±0.01	0.89±0.02	0.80±0.00	0.51±0.01	1.47±0.01	1.02±0.03	0.41±0.01	1.00±0.01	0.86±0.01	0.90±0.01	0.51±0.01	0.80±0.01	0.75±0.01	1.05±0.01	1.19±0.01	1.03±0.00
Phenylethyl acetate (mg/mL)	0.40±0.00	0.75±0.05	0.56±0.03	0.20±0.01	0.53±0.01	0.53±0.03	0.44±0.01	0.45±0.00	0.43±0.01	0.48±0.02	0.68±0.01	0.44±0.02	0.49±0.00	0.47±0.04	0.40±0.05	0.36±0.02
Ethyl hexanoate (mg/mL)	0.67±0.01	0.74±0.00	0.72±0.02	0.59±0.01	0.91±0.01	0.77±0.02	0.54±0.06	0.65±0.01	0.31±0.04	0.53±0.01	0.40±0.00	0.65±0.00	0.88±0.03	0.81±0.01	0.94±0.01	0.46±0.01
Ethyl butyrate (mg/mL)	ND	0.06±0.00	0.06±0.00	0.02±0.00	ND	0.06±0.01	0.05±0.01	0.12±0.00	0.09±0.00	0.04±0.00	ND	0.04±0.00	ND	ND	ND	ND
Ethyl octanoate (μg/mL)	2.52±0.00	4.00±0.07	2.51±0.18	3.35±0.58	5.74±0.40	4.28±0.44	3.73±0.02	2.28±0.18	3.07±0.09	3.69±0.19	3.36±0.09	2.06±0.25	5.25±0.16	3.24±0.11	5.18±0.09	4.26±0.02
Diethyl succinate (mg/mL)	0.05±0.00	0.05±0.00	0.01±0.00	0.03±0.00	0.04±0.00	0.03±0.00	0.02±0.00	0.01±0.00	0.01±0.00	0.03±0.00	0.05±0.00	0.05±0.00	0.04±0.00	0.03±0.00	0.03±0.00	0.05±0.00
Ethyl acetate (mg/mL)	13.04±0.14	8.07±0.20	9.17±0.55	5.28±0.48	14.99±1.04	11.65±0.82	6.87±0.05	13.20±0.13	3.96±0.03	14.24±0.19	15.39±0.27	18.80±0.35	10.37±0.52	10.91±0.06	11.75±0.21	5.79±0.40
**Terpenes**																
Linalol (μg/mL)	3.36±0.25	8.02±1.82	2.20±0.03	31.72±5.74	23.85±1.29	20.35±1.94	21.35±0.51	5.65±1.20	11.68±1.83	7.55±1.29	17.73±0.75	6.46±0.96	5.81±0.41	5.49±0.47	ND	3.63±0.14
Nerol (μg/mL)	4.05±0.35	4.95±0.54	6.15±0.78	2.38±0.12	2.59±0.11	2.24±0.17	2.37±0.37	2.12±0.22	4.47±0.12	4.41±0.22	4.55±0.79	7.09±0.60	5.11±0.22	5.18±0.23	2.58±0.42	5.53±0.53
Geraniol (μg/mL)	3.33±0.05	7.59±1.40	6.52±0.51	4.84±0.19	3.24±0.10	3.88±0.14	4.67±0.14	7.11±0.84	5.34±0.03	7.07±0.19	8.59±0.31	4.98±0.28	4.89±0.16	4.35±0.07	4.53±0.07	4.31±0.06

**Enones**																	
β-Damascenone (μg/mL)	2.42±0.21	6.56±0.37	2.57±0.04	5.42±0.95	2.99±0.00	4.89±0.08	8.09±1.92	3.62±1.98	5.54±0.06	5.53±0.41	4.11±0.43	4.71±0.23	1.75±0.06	3.51±0.39	2.64±0.06	5.91±0.16

*= Biotypes X,XI and XII were not evaluated due to the lost during the recovery after conservation.

**= Not detected
